# Analysis of factors affecting outcome in acute type A aortic dissection complicated by preoperative cardiopulmonary resuscitation

**DOI:** 10.1093/ejcts/ezad436

**Published:** 2024-01-04

**Authors:** Matteo Montagner, Markus Kofler, Leonard Pitts, Simone Gasser, Lukas Stastny, Stephan D Kurz, Michael Grimm, Volkmar Falk, Jörg Kempfert, Julia Dumfarth

**Affiliations:** Department of Cardiothoracic and Vascular Surgery, Deutsches Herzzentrum der Charité (DHZC), Berlin, Germany; Charité—Universitätsmedizin Berlin, corporate member of Freie Universität Berlin, Humboldt-Universität zu Berlin, and Berlin Institute of Health, Berlin, Germany; Department of Cardiothoracic and Vascular Surgery, Deutsches Herzzentrum der Charité (DHZC), Berlin, Germany; Charité—Universitätsmedizin Berlin, corporate member of Freie Universität Berlin, Humboldt-Universität zu Berlin, and Berlin Institute of Health, Berlin, Germany; DZHK (German Center for Cardiovascular Research), partner site Berlin, Berlin, Germany; Department of Cardiothoracic and Vascular Surgery, Deutsches Herzzentrum der Charité (DHZC), Berlin, Germany; Charité—Universitätsmedizin Berlin, corporate member of Freie Universität Berlin, Humboldt-Universität zu Berlin, and Berlin Institute of Health, Berlin, Germany; University Clinic of Cardiac Surgery, Innsbruck Medical University, Innsbruck, Austria; University Clinic of Cardiac Surgery, Innsbruck Medical University, Innsbruck, Austria; Department of Cardiothoracic and Vascular Surgery, Deutsches Herzzentrum der Charité (DHZC), Berlin, Germany; Charité—Universitätsmedizin Berlin, corporate member of Freie Universität Berlin, Humboldt-Universität zu Berlin, and Berlin Institute of Health, Berlin, Germany; University Clinic of Cardiac Surgery, Innsbruck Medical University, Innsbruck, Austria; Department of Cardiothoracic and Vascular Surgery, Deutsches Herzzentrum der Charité (DHZC), Berlin, Germany; Charité—Universitätsmedizin Berlin, corporate member of Freie Universität Berlin, Humboldt-Universität zu Berlin, and Berlin Institute of Health, Berlin, Germany; DZHK (German Center for Cardiovascular Research), partner site Berlin, Berlin, Germany; Translational Cardiovascular Technologies, Institute of Translational Medicine, Department of Health Sciences and Technology, Swiss Federal Institute of Technology (ETH) Zurich, Switzerland; Department of Cardiothoracic and Vascular Surgery, Deutsches Herzzentrum der Charité (DHZC), Berlin, Germany; Charité—Universitätsmedizin Berlin, corporate member of Freie Universität Berlin, Humboldt-Universität zu Berlin, and Berlin Institute of Health, Berlin, Germany; DZHK (German Center for Cardiovascular Research), partner site Berlin, Berlin, Germany; University Clinic of Cardiac Surgery, Innsbruck Medical University, Innsbruck, Austria

**Keywords:** Cardiopulmonary resuscitation, Type A aortic dissection, Risk factor analysis, Outcome

## Abstract

**OBJECTIVES:**

Cardiopulmonary resuscitation (CPR) aggravates the pre-existing dismal prognosis of patients suffering from acute type A aortic dissection (ATAAD). We aimed to identify factors affecting survival and outcome in ATAAD patients requiring CPR at presentation at 2 European aortic centres.

**METHODS:**

Data on 112 surgical candidates and undergoing preoperative CPR were retrospectively evaluated. Patients were divided into 2 groups according to 30-day mortality. A multivariable model identified predictors for 30-day mortality.

**RESULTS:**

Preoperative death occurred in 23 patients (20.5%). In the remaining 89 surgical patients (79.5%) circulatory arrest time (41 ± 20 min in 30-day non-survivors vs 30 ± 13 min in 30-day survivor, *P* = 0.003) as well as cardiopulmonary bypass time (320 ± 132 min in 30-day non-survivors vs 252 ± 140 min in 30-day survivor, *P* = 0.020) time was significantly longer in patients with worse outcome. Thirty-day mortality of the total cohort was 61.6% (*n* = 69) with cardiac failure in 48% and aortic rupture or haemorrhagic shock (28%) as predominant reasons of death. Age [odds ratio (OR) 1.04, 95% confidence interval (CI) 1.01–1.09, *P* = 0.034], preoperative coronary (OR 3.42, 95% CI 1.34–9.26, p = 0.012) and spinal malperfusion (OR 12.49, 95% CI 1.83–225.02, *P* = 0.028) emerged as independent predictors for 30-day mortality while CPR due to tamponade was associated with improved early survival (OR 0.29, 95% CI 0.091–0.81, *P* = 0.023).

**CONCLUSIONS:**

Assessment of underlying cause for CPR is mandatory. Pericardial tamponade, rapidly resolved with pericardial drainage, is a predictor for improved survival, while age and presence of coronary and spinal malperfusion are associated with dismal outcome in this high-risk patient group.

**Figure:**
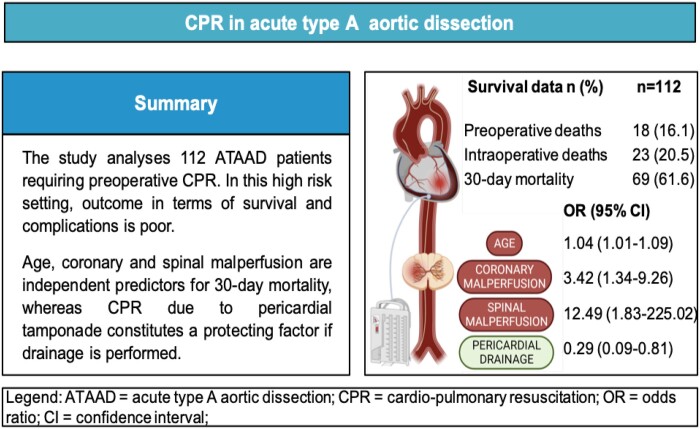

## INTRODUCTION

Acute type A aortic dissection (ATAAD) is a lethal condition with dismal outcomes without surgical intervention. Complex dissection morphologies and preoperative clinical complications need to be addressed in a timely fashion as they add an additional complexity level to emergent surgical repairs. ATAAD is aggravated by cardiopulmonary arrest (CPA) necessitating cardiopulmonary resuscitation (CPR) in 3.4% and 6.6% of all patients [1–3]. CPR at presentation was demonstrated as independent predictor for mortality in the setting of ATAAD [4]. Limited literature is available on this topic, mainly based on case reports and relatively small case series on a maximum of 44 patients [1–3, 5]. This subgroup of patients experiences extremely high mortality rates up to 61.8% compared to the overall ATAAD population [3]. The goal of this study was to investigate factors affecting survival and outcome in ATAAD patients requiring CPR at presentation that were deemed as surgical candidates at 2 European aortic centres.

## PATIENTS AND METHODS

### Ethics statement

The study was approved by local ethics committee in both centres (Charité Medical School, Berlin, Germany, No. EA2/096/20; Medical University Innsbruck, Innsbruck, Austria No. UN5106) and complies with the Declaration of Helsinki. Patient informed consent was waived based on the retrospective character of the study.

### Patient population

Patients suffering from ATAAD between January 2000 and March 2022 from 2 high-volume centres were screened (*n* = 1997). Out of these, 112 patients (5.6%)—deemed surgical candidates despite undergoing preoperative CPR before or after diagnosis of ATAAD was confirmed with computed tomography (CT) scans—were retrospectively included in this study. CPR was carried out in-hospital by trained medical staff in the majority of cases (83.9%), while 16.1% were initially resuscitated by lay rescuers or paramedics. In 2 patients undergoing CPR with suspected coronary ischaemia, venoarterial extracorporeal membrane oxygenation (vaECMO) was implanted under radiographic guidance before ATAAD was confirmed with CT. Clinical data as well as imaging studies were evaluated. Coronary malperfusion was defined as ischaemia-specific pathological findings in electrocardiogram with or without wall motion abnormalities or elevation of cardiac enzymes. Aortic rupture was defined as abrupt haemodynamic collapse concomitant with massive pericardial effusion that was absent in the CT scan or in the echocardiography, which led to emergency connection to cardiopulmonary bypass (CPB) or rapid exsanguination and was confirmed intraoperatively. Arrhythmogenic events were defined in the few cases where no coronary malperfusion or tamponade were present, but still the patient required CPR after developing malignant arrhythmias. Spinal malperfusion was defined as a new onset of paraplegia. According to 30-day mortality, which was defined as death within the first 30 days after diagnosis of ATAAD, patients were divided into 2 groups (30-day survivor and 30-day non-survivor).

### Surgical repair

Once diagnosis of ATAAD was confirmed, patients were immediately transferred and rushed to the operation theatre. Out of 112 patients, 23 patients (20.5%) passed away, either during induction of anaesthesia (*n* = 18) or before CPB (*n* = 5) could successfully be installed. In the remaining 89 patients (79.5%), surgical repair was performed. Operative strategy has been previously described [6]. Due to the critical state of these patients, rapid arterial cannulation was predominantly performed via femoral artery (*n* = 55, 61.8%), and axillary artery was cannulated in slightly stabilized patients (*n* = 27, 29.2%). Direct cannulation of the aorta (*n* = 6; 6.7%) or cannulation of the innominate trunk (*n* = 2; 2.2%) was chosen less frequently.

The majority of surgical repairs were performed in hypothermic circulatory arrest (HCA) (86.5%) with a mean circulatory arrest time of 36 ± 17 min. Antegrade cerebral perfusion with cold blood (20–25°C) at a flow rate of 10–15 ml/kg/min body weight was used in 38 patients (41.3%). Isolated retrograde cerebral perfusion (*n* = 36; 39.1%) via an angled cannula, which was inserted in the superior vena cava and snared during HCA, was utilized mainly in the early study period (2000–2009). Straight deep HCA without additional cerebral perfusion was performed in very selected cases (*n* = 5; 5.4%).

A primarily tear-oriented approach towards surgical repair was followed in both surgical centres. Depending on the extent of intimal defects or a pre-existing dilatation, root (*n* = 31, 34.8%) and/or total arch replacement (*n* = 11; 12.4%) were performed. Additional coronary artery bypass grafting (CABG) was performed in 25 patients with either coronary disruption or the presence of severe calcification and myocardial ischaemia. Postoperative treatment was standardized for every patient at a dedicated, experienced intensive care unit.

### Statistical analysis

Statistical analysis was performed using SPSS (IBM Corp. Released 2021. IBM SPSS Statistics for Windows, Version 28.0. Armonk, NY: IBM Corp) and R Version 4.0.0 [R Development Core Team (2019). R: A Language and Environment for Statistical Computing]. Categorical variables are presented as frequencies with corresponding percentages. Continuous variables were expressed as mean ± standard deviation. Differences between the 2 groups were tested by means of Chi-square test or Fisher’s exact test (in case of expected cell frequencies were <5) or Student’s *t*-test as appropriate.

In order to identify the multivariable binary logistic regression model with the best fit, stepwise backward variable selection based on Akaike’s information criterion was performed to identify independent associates of 30-day mortality. To test the model assumptions, the variance inflation factor was used. A variance inflation factor of <5 was considered to rule out a significant effect on the model induced by multi-collinearity. Results of regression analyses were displayed as odds ratio (OR) with corresponding 95% confidence interval (CI). A *P*-value of <0.05 was considered as statistically significant. Mortality rates were calculated using the Kaplan–Meier method and compared using log-rank test. Kaplan–Meier curves were drawn using R version 3.6.0 (Library ‘Survival’ and ‘Survminer’).

## RESULTS

### Preoperative management and demographics

The study group consisted of 112 patients with a mean age of 63.4 ± 12 years. CPR was predominantly performed by trained medical staff in-hospital (83.9%). Reason for CPR was grouped in 4 different entities, with cardiac tamponade as the leading cause (40.2%), followed by coronary malperfusion (26.8%), aortic rupture (19.6%) or arrhythmogenic events (13.4%). All patients presented in a very critical state with a calculated German registry of acute aortic dissection type A score of 64%. Coronary malperfusion was significantly higher in patients with dismal outcome (30-day non-survivors 64.7% vs 30-day survivors 41.9%, *P* = 0.018). While rapid diagnosis and transfer to the operation theatre were provided in both centres, mortality rate prior to successful installation of CPB predominantly due to aortic rupture was high (20.5%). Further details are presented in Table 1.

**Table 1:** Baseline characteristics

	Total cohort, *n* = 112	30-Day survivors, *n* = 43	30-Day non-survivors, *n* = 69	*P*-value
Gender (female)	53 (47)	21 (49)	32 (46)	0.80
Age (years)	63 ± 12	62 ± 12	64 ± 12	0.42
Previous cardiac surgery	3 (2.7)	2 (5)	1 (2)	0.31
Preoperative intubation	72 (64.3)	26 (61)	46 (67)	0.51
Acute preoperative neurologic deficit	45 (40.2)	16 (37)	29 (43)	0.57
Cardiac tamponade	65 (59.6)	24 (56)	41 (62)	0.51
Moderate to severe aortic regurgitation	35 (38)	13 (33)	22 (43)	0.30
Preoperative malperfusion	84 (75)	29 (67)	55 (80)	0.15
Coronary	62 (55.4)	18 (42)	44 (65)	**0.018**
Cerebral	37 (33)	13 (30)	24 (35)	0.58
Spinal	9 (8)	1 (2)	8 (12)	0.15
Extremity	18 (16.1)	7 (16)	11 (16)	0.99
Renal	9 (8)	4 (9)	5 (7)	0.73
Abdominal	15 (13.4)	3 (7)	12 (18)	0.16
Penn classification				
Aa class	16 (14.3)	7 (16)	9 (13)	0.63
Ab class	10 8.9)	7 (16)	3 (4)	**0.031**
Ac class	33 29.5)	13 (30)	20 (29)	0.89
Abc class	53 (47.3)	16 (37)	37 (54)	0.09
GERAADA score (%)	64 ± 16	60 ± 16	66 ± 16	0.11
CPR place				
In-hospital	94 (83.9)	36 (84)	58 (84)	0.96
Aortic centre CPR	67 (59.8)	23 (54)	44 (64)	0.24
CPR reason				
Tamponade	45 (40.2)	21 (49)	24 (35)	0.14
Coronary malperfusion	30 (26.8)	11 (4)	19 (28)	0.82
Aortic rupture	22 (19.6)	5 (12)	17 (25)	0.092
Arrhythmogenic events	15 (13.4)	6 (14)	9 (13)	0.89
Extracoropeal CPR via vaECMO	2 (1.8)			
Death before CPB installation	23 (20.5)			

Data are displayed as *n* (%) or mean ± SD.

CPB: cardiopulmonary bypass; CPR: cardiopulmonary resuscitation; GERAADA: German registry of acute aortic dissection type A; SD: standard deviation; vaECMO: venoarterial extracorporeal membrane oxygenation.

Statistical significant values (*p*<0.05) are displayed in bold.

### Surgical repair

Pain-to-cut time was 520 ± 436 min, with no difference between 30-day survivors and 30-day non-survivors. There was a significantly higher rate of right axillary cannulation in 30-day survivors (40% vs 30-day non-survivor 20%, *P* = 0.038). While extent of surgical repair did not differ between the groups, circulatory arrest time (41 ± 20 min in 30-day non-survivors vs 30 ± 13 min in 30-day survivor, *P* = 0.003) as well as CPB (320 ± 132 min in 30-day non-survivors vs 252 ± 140 min in 30-day survivor, *P* = 0.020) time was significantly longer in patients with worse outcome. Almost 1 out of 4 patients in the 30-day non-survivor group needed mechanical support due to severe impairment of left ventricular function (intraoperative vaECMO implantation; 30-day non-survivors 24% vs 30-day survivors 2%, *P* = 0.003). Table 2 provides detailed surgical data.

**Table 2:** Operative data

	Total cohort, *n* = 89	30-Day survivors, *n* = 43	30-Day non-survivors, *n* = 46	*P*-value
Pain-to-cut time (min)	520 ± 436	567 ± 467	468 ± 399	0.34
Cardiopulmonary bypass time (min)	287 ± 139	252 ± 140	320 ± 132	**0.020**
Cross-clamping time (min)	124 ± 54	116 ± 54	132 ± 54	0.17
Circulatory arrest time (min)	36 ± 17	30 ± 13	41 ± 20	**0.003**
Lowest operative temperature (°C)	21.5 ± 6.3	22 ± 6	21 ± 6	0.34
Primary arterial cannulation site				
Aorta	6 (7)	1 (2)	5 (11)	0.11
Innominate artery	2 (2)	0 (0)	2 (4)	0.17
Right axillary artery	27 (29)	17 (40)	9 (20)	**0.038**
Femoral artery	55 (62)	25 (58)	30 (66)	0.49
Open distal anastomosis	77 (87)	37 (86)	40 (87)	0.90
Cerebral perfusion strategy				
DHCA alone	5 (5)	3 (7)	2 (4)	0.67
Retrograde cerebral perfusion	36 (39)	15 (35)	21 (46)	0.30
Antegrade cerebral perfusion	38 (41)	21 (49)	17 (37)	0.26
Root replacement	31 (35)	14 (33)	17 (37)	0.66
Arch replacement	11 (12)	3 (7)	8 (17)	0.14
Concomitant CABG	23 (26)	10 (23)	13 (28)	0.59
Intraoperative vaECMO implantation	12 (14)	1 (2)	11 (24)	**0.003**
In-hospital mortality	71 (63)			

Data are displayed as *n* (%) or mean ± SD.

CABG: coronary artery bypass grafting; DHCA: deep hypothermic circulatory arrest; SD: standard deviation; vaECMO: veno-arterial extracorporeal membrane oxygenation support.

Statistical significant values (*p*<0.05) are displayed in bold.

### Risk factors for 30-day mortality

Overall 30-day mortality in patients undergoing CPR was high with a rate of 61.6% (*n* = 69). The predominant reason of death was cardiac failure in 48%, followed by aortic rupture or haemorrhagic shock (28%) and neurologic complications in 15%. Multiorgan failure, sepsis or a combination of both accounted for <10%.

Multivariable regression analysis identified age (OR 1.04, 95% CI 1.01–1.09, *P* = 0.034), preoperative coronary malperfusion (OR 3.42, 95% CI 1.34–9.26, *P* = 0.012) and spinal malperfusion (OR 12.49, 95% CI 1.83–225.02, *P* = 0.028) as independent predictors for 30-day mortality while CPR due to tamponade was associated with improved early survival (OR 0.29, 95% CI 0.091–0.81, *P* = 0.023) (Table 3).

**Table 3:** Multivariable regression analysis to identify variables associated with 30-day mortality

	Univariable		Multivariable		VIF
	OR [95% CI]	*P*-value	OR [95% CI]	*P*-value	
**Age (years)**	1.01 [0.98–1.05]	0.42	**1.04 [1.01–1.09]**	**0.034**	1.1
**Coronary malperfusion**	2.44 [1.13–5.40]	0.025	**3.42 [1.34–9.26]**	**0.012**	1.2
**Spinal malperfusion**	5.51 [0.96–104.12]	0.11	**12.49 [1.83–225.02]**	**0.028**	1.1
Abdominal malperfusion	2.81 [0.83–12.90]	0.13	4.33 [1.09–22.97]	0.05	1.1
**CPR due to tamponade**	0.56 [0.25–1.21]	0.14	**0.29 [0.09–0.81]**	**0.023**	1.4
CPR due to coronary dissection	1.11 [0.47–2.69]	0.82	0.37 [0.10–1.22]	0.11	1.5

CI: confidence interval; CPR: cardiopulmonary resuscitation; OR: odds ratio; VIF: variance inflation factor.

Statistical significant values (*p*<0.05) are displayed in bold.

### Postoperative outcome

Out of 89 patients undergoing surgical repair, 26 patients died intraoperatively or within the first 24 h postoperatively. Outcome analysis was performed in patients exceeding this period (*n* = 63) (Table 4). Because the non-survivors group was more likely to undergo discontinuation of therapy, length of intensive care unit stay was significantly shorter in these patients. Overall postoperative morbidity was high with prolonged intubation and increased rates of continuous renal replacement therapy (46%). 41% of patients suffered from perioperative stroke with permanent neurologic deficit at discharge or transfer to neurologic rehab clinics in 94%. Functional follow-up of 26 patients surviving 1 year or longer revealed good recovery in 18 patients, persistent neurology but ability to walk with mobility aids in 5 patients and impaired neurologic recovery and wheelchair dependency in 3 patients.

**Table 4:** Perioperative outcome exceeding 24 h postoperatively

	Total cohort, *n* = 63	30-Day survivors, *n* = 43	30-Day non-survivors, *n* = 20	*P*-value
ICU length of stay (days)	16 ± 16	25 ± 16	6 ± 5	**<0.001**
Revision for bleeding	21 (33)	14 (33)	7 (35)	0.85
Ventilation >48 h	53 (84)	39 (91)	14 (70)	0.061
Postoperative CRRT	29 (46)	17 (40)	12 (60)	0.13
Postoperative stroke	26 (41)	18 (42)	8 (40)	0.89

Data are displayed as *n* (%) or mean ± SD.

CRRT: continuous renal replacement therapy; ICU: intensive care unit.

Statistical significant values (*p*<0.05) are displayed in bold.

**Figure 1:**
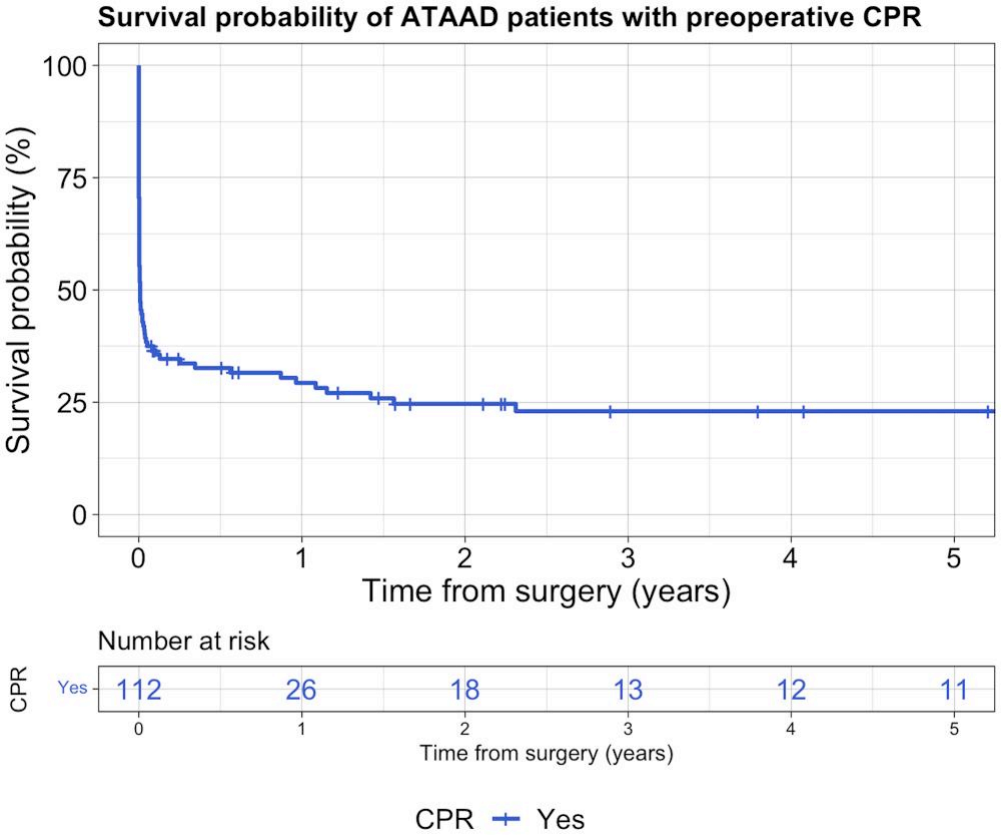
Survival probability following CPR. CPR: cardiopulmonary resuscitation.

Despite tremendous early mortality, survival after discharge was rather stable with 29.3 ± 4% at 1 year and 22 ± 4% at 3 and 5 years (Fig. 1).

## DISCUSSION

**Figure 2:**
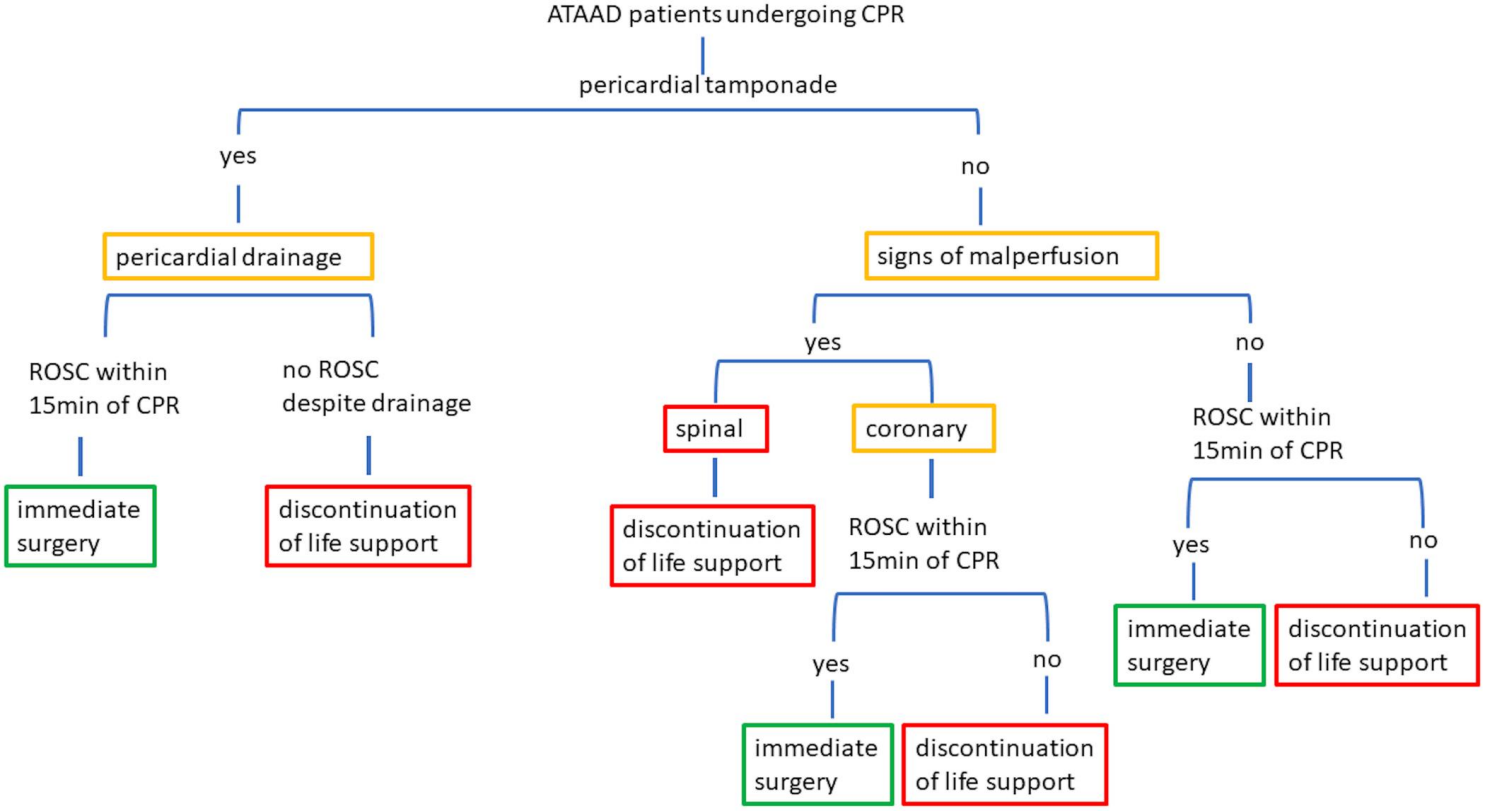
Treatment algorithm following CPR. CPR: cardiopulmonary resuscitation.

The goal of this paper was to investigate factors affecting outcomes in patients suffering ATAAD with preoperative CPR that were accepted for surgery at 2 aortic centres. Our data show high mortality rates (30-day mortality 61.6%), with age, coronary and spinal malperfusion being independent predictors for mortality. Nevertheless, patients undergoing CPR due to cardiac tamponade were more likely to experience favourable outcomes. According to these findings and the available literature on the duration of CPR, we propose an algorithm that may aid the decision-making when treating such a high-risk subgroup of patients (Fig. 2).

Enhanced awareness of the disease and improvements in diagnostics and treatment improved survival rates of ATAAD over the last 2 decades [7–9]. At the same time, this evolution confronts us with a more demanding and complex patient cohort and the evident need for optimized preoperative risk assessment. CPA requiring CPR reflects the most urgent condition where a choice between escalation of therapy and proceeding to surgical treatment or discontinuation of life-saving processes has to be made in ATAAD patients. Necessity of preoperative CPR has been proven as independent risk factor for impaired survival [3, 10]. This has been reflected in preoperative mortality risk calculation tools like the German registry of acute aortic dissection type A score, where preoperative CPR accounts for the highest OR for 30-day mortality [11]. There seems to be a subgroup of patients who benefit from surgery that ideally could be identified by preoperative markers.

When it comes to the prevalence of CPR in ATAAD, registry data as well as experienced centres from all around the world report rates ranging from 3.4% to 6.6% [1, 3]. This is very much in line with our reported CPR rate of 5.6%. Interestingly, data from Japan recently reported diverging results with an extremely high rate of preoperative CPA in ATAAD patients of 33.1% [12]. In their study cohort, a well-defined institutional protocol for extracorporeal cardiopulmonary resuscitation (eCPR) was followed and vaECMO was implanted within a very short time interval from collapse in 31 patients not responding to conventional CPR. Twenty-four patients died before surgery mainly due to aortic rupture, while 7 patients were bridged to surgery but also had dismal outcome. Ohbe *et al.* provided outcome data from the Japanese Diagnosis Procedure Combination inpatient database on 398 patients undergoing eCPR in the setting of ATAAD and showed a 98% mortality rate. In addition, eCPR was associated with an incremental economic burden of 161 504 US Dollars per quality-adjusted life year gained [13]. In our study cohort, eCPR via femoro-femoral vaECMO support was applied in 2 patients with CPA and primarily suspected acute coronary syndrome. After correct diagnosis of ATAAD was confirmed in these patients, surgical repair was proceeded due to young age, but both patients died within 48 h after surgery. While eCPR might stabilize the patient in terms of organ perfusion, it can trigger or worsen lethal aortic rupture, which accounted for high mortality in the study by Nakai and colleagues [12]. In our 2 patients, we assume that retrograde flow of vaECMO from femoral cannulation worsened myocardial perfusion due to extensive perfusion of the false lumen via the entry tear. Based on the existing evidence and the limited numbers provided in literature, one might conclude that eCPR should not be considered in the setting of CPA and confirmed ATAAD.

Even if rapid decision has to be made once patients with ATAAD code and need CPR, a basic understanding of the underlying reason for CPA is mandatory in order to define next steps in patient management. Cardiac tamponade was the most common cause for CPR in our cohort and other series [1–3]. This underlying cause of severe haemodynamic compromise can be treated rapidly with pericardial drainage. One has to keep in mind, that performance of pericardial drainage should not delay surgery. According to the 2010 Guidelines of the AAC/American Heart Association/American Association of Thoracic Surgeons, pericardiocentesis can be performed in this patient cohort who cannot survive until surgery by withdrawing just enough fluid to restore perfusion [14]. This recommendation has been also promoted in the most recent expert consensus statement of the American Association for Thoracic Surgery [15]. Pericardial tamponade reflects a life-threatening condition that can be resolved rapidly in experienced hands and can lead to an immediate improvement of haemodynamics. Nakai *et al.* [12] reported significantly higher survival rates in patients undergoing urgent pericardial drainage. This underlines our findings that pericardial tamponade emerged as the only preoperative predictor for improved survival in our patient cohort.

Risk factor analysis for early mortality in a surgical cohort of patients undergoing preoperative CPR is limited due to the low number of patients ranging from 17 to a maximum of 44 recently reported from the NORCAAD registry [5, 13]. This study provides the so far largest cohort of 89 patients undergoing emergent surgery after CPR in ATAAD. Risk factor analysis revealed age and spinal as well as coronary malperfusion as important associates for early mortality. Age is a well-known risk factor for impaired survival in a surgical ATAAD patient group [11]. Complicated type A dissection, defined as presence of malperfusion, neurologic injury or CPR, emerged as independent risk factor for 30-day survival in octogenarians in one of our past studies [16]. Coronary malperfusion as another important risk factor has been extensively studied. Preoperative coronary malperfusion is associated with a higher operative mortality up to 41.5% [17, 18]. Hashimoto and colleagues illustrated in a multivariable model, that cardiac ischaemia in combination with cardiogenic shock (Killip class IV) was a strong predictor for in-hospital mortality (OR 2.86, 95% CI 1.50–5.44) [18]. Preoperative coronary malperfusion as independent predictor for early mortality was reconfirmed in our cohort. Despite a rapid approach towards surgical treatment, coronary ischaemia and hypoperfusion due to CPR was associated with irreversible myocardial damage in most patients being reflected in the high rate of vaECMO support. Only 1 out of 12 patients being bridged with intraoperative implantation of vaECMO survived. Identical data on dismal vaECMO outcome were presented by Uehara and colleagues [3]. Outcome of patients with spinal malperfusion and CPR in our cohort was disillusioning with 100% mortality rate within the first 30 days.

While we have to accept that overall mortality rate is extremely high in patients suffering from ATAAD and undergoing CPR, we intended to identify patients who still might benefit from surgical repair. Our database did not depict reliable documentation of CPR duration; therefore, we were unable to consider this important factor in our risk assessment. In literature, there is strong evidence that CPR duration exceeding 15 min is associated with an 8-fold increase in-hospital mortality [3].

### Limitations

Major limitations in this study originate from its retrospective nature. As data from 2 aortic centres with standardized patient care were merged, local differences especially pre-hospital treatment algorithms might have affected the outcome. Despite the fact that most patients underwent CPR in professional hands, duration of CPR was not sufficiently documented and therefore could not be included in our data presentation. In patients undergoing CPR detailed preoperatively neurologic evaluation is limited due to the emergency situation and the need for sedation and intubation. Our registries do not cover patients undergoing CPR in the setting of ATAAD being denied from surgery, this unreported number of patients would be of great interest. Furthermore, the logistic regression model and the resulting cocnlusions need to be interpreted under the light of the low event rate of certain variables. This is especially the case for spinal malperfusion.

## CONCLUSION

Patients undergoing CPR in the setting of ATAAD have to be carefully evaluated for the reason of CPA. Pericardial tamponade, which can rapidly be resolved with pericardial drainage is a predictor for improved survival, while age and presence of coronary and spinal malperfusion are associated with dismal outcome in this high-risk patient group.

## Data Availability

The data obtained for this manuscript cannot be shared publicly due to data protection policies but will be provided to interested parties upon reasonable request to the corresponding author.

## Notes

Presented at the EACTS Annual Meeting 2022, Milan, Italy.

## ABBREVIATIONS

ATAAD: Acute type A aortic dissection
CPB: Cardiopulmonary bypass
CI: Confidence interval
CPA: Cardiopulmonary arrest
CPR: Cardiopulmonary resuscitation
CT: Computed tomography
eCPR: Extracorporeal cardiopulmonary resuscitation
HCA: Hypothermic circulatory arrest
OR: Odds ratio
vaECMO: Venoarterial extracorporeal membrane oxygenation
